# Comprehensive care for patients with Chagas cardiomyopathy during the coronavirus disease pandemic

**DOI:** 10.1590/0037-8682-0353-2020

**Published:** 2020-10-05

**Authors:** Flavia Mazzoli-Rocha, Fernanda de Souza Nogueira Sardinha Mendes, Paula Simplicio Silva, Gilberto Marcelo Sperandio da Silva, Mauro Felippe Felix Mediano, Andréa Silvestre de Sousa

**Affiliations:** 1 Fundação Oswaldo Cruz, Instituto Nacional de Infectologia Evandro Chagas, Rio de Janeiro, RJ, Brasil.

Dear Editor:

Chagas disease is a neglected tropical disease according to the World Health Organization^1^. The most common clinical complication of Chagas disease is Chagas cardiomyopathy, that represents the main cause of non-ischemic cardiomyopathy in Latin America, affecting 20% to 40% of infected people^2^. The chronic form, with a slow and persistent course, results from the destruction of the myocardial fibers, caused by a chronic inflammatory process. It is associated with intense reparative fibrosis and progressive ventricular remodeling and manifests as heart failure and/or arrhythmic and thromboembolic syndromes^3^.

The elevated morbimortality related to Chagas cardiomyopathy^1^ and affected population profiles of low income and poor education directly impact education, interfering with self-care and adherence to treatment programs^4^. In this setting, cardiovascular rehabilitation, which improves the functional capacity in chronic Chagas cardiomyopathy^5^ ^-^ ^8^, seems to be a favorable place to institute self-care strategies and adherence to treatment programs^4^. 

To improve the care of patients with cardiomyopathy, many types of remote assessments have garnered attention in the past decades^9^ ^,^ ^10^. The American Association of Cardiovascular and Pulmonary Rehabilitation considers that healthcare professionals should adhere to the practice of cardiopulmonary rehabilitation through remote assessments to assure the highest quality of patient care^11^.

More studies are required to better conclude the effects of remote assessment strategies on the outcome of patients with cardiomyopathy. For example, telemonitoring and structured telephone support seem to reduce hospitalizations and mortality in patients with cardiovascular diseases^10^. Moreover, the improvement in functional capacity does not differ for patients with chronic heart failure subjected to telerehabilitation compared to a center-based program^12^. To the best of our knowledge, there have been no studies on remote assessments in the management of Chagas cardiomyopathy. However, considering the importance of social isolation during the coronavirus disease (COVID-19) pandemic and need for a close follow-up for most patients with Chagas cardiomyopathy, the remote assessment strategy emerges as a useful alternative during this pandemic period in order to ensure continued comprehensive care delivered to patients while complying with social distancing^13^.

The healthcare team from the Evandro Chagas National Institute of Infectious Disease, a national reference center for the diagnosis and treatment of infectious diseases that regularly follows-up more than 1,000 patients with chronic Chagas disease under a comprehensive care treatment, started a telephone support for all patients with chronic Chagas cardiomyopathy enrolled in the cardiovascular rehabilitation program before the COVID-19 pandemic. To start the telephone support, a questionnaire was established (Table 1), and a service scale was organized to contact patients to obtain information about their healthcare guidance, answer questions, and provide general guidance on healthcare during the pandemic. During the contact, the health professional confirmed the date of the next medical appointment or need for a new one and filled-out the questionnaire, obtaining information about weight control and blood glucose, regularity of food and drink intake, treatment adherence, personal hygiene measures, presence of physical and mental symptoms, financial difficulty, and maintenance of social isolation. To evaluate treatment adherence, based on our previous experience^14^, questions were asked to know if patients forgot to take medication, if they took it at the correct time, and if they stopped the medication because they felt better or worse. In relation to personal hygiene measures, we asked about the ability to perform activities of daily living independently, including the ability to eat, dress, and shower without any help. To evaluate physical and mental symptoms, questions about the presence of breathlessness, pain, heart palpitations, fainting episodes, sadness, and anxiety were asked. Despite being a simple telephone contact, patients demonstrated satisfaction with the team concerned, and it was possible to collect the general information about their clinical status, such as significant weight change and the presence of symptoms. Finally, we believe that this telephone support strategy may help at least in part the maintenance of comprehensive care for our patients and could alert us for early signs and symptoms of decompensation, providing the opportunity for intervention before patients require hospitalization.

**TABLE 1:** Questions to be answered routinely by patients during isolation due to the coronavirus disease pandemic.

	Questionnaire	
1	Are you feeling good?	0/No ( ); 1/Yes ( )
2	Do you ever forget to take your medicine?	0/No ( ); 1/Yes ( )
3	Are you careless at times about taking your medicine?	0/No ( ); 1/Yes ( )
4	When you feel better do you sometimes stop your medicine?	0/No ( ); 1/Yes ( )
5	If sometimes you feel worse when you take the medicine, do you stop taking it?	0/No ( ); 1/Yes ( )
6	Are you maintaining your body weight?	0/No ( ); 1/Yes ( ); Last weight: ___
7	Have you been eating well?	0/No ( ); 1/Yes ( )
8	Have you controlled your fluid intake?	0/No ( ); 1/Yes ( )
9	Have you checked your glucose daily?	0/No ( ); 1/Yes ( ); Last glucose: ___
10	Are you managing to take care of your hygiene without help?	0/No ( ); 1/Yes ( )
11	Have you been active at home?	0/No ( ); 1/Yes ( )
12	Are you taking care of children or grandchildren at home?	0/No ( ); 1/Yes ( )
13	Have you experienced breathlessness at bedtime?	0/No ( ); 1/Yes ( )
14	Have you had chest pain, a racing heart, or a fainting episode?	0/No ( ); 1/Yes ( )
15	Are you sad from staying at home?	0/No ( ); 1/Yes ( )
16	Are you feeling anxious?	0/No ( ); 1/Yes ( )
17	Are you having financial difficulties?	0/No ( ); 1/Yes ( )
18	Do you live alone?	0/No ( ); 1/Yes ( )
19	Do you have any questions about your illness?	0/No ( ); 1/Yes ( )
20	Have you received the influenza vaccine?	0/No ( ); 1/Yes ( )
21	Do you understand the importance of social isolation?	0/No ( ); 1/Yes ( )

## References

[1] Dias JCP, Junior ANR, Gontijo ED, Luquetti A, Shikanai-Yasuda MA, Coura JR (2016). II Consenso Brasileiro em Doença de Chagas, 2015. Epidemiol Serv Saude.

[2] Benziger CP, Carmo GA, Ribeiro AL (2017). Chagas Cardiomyopathy: Clinical Presentation and Management in
the Americas. Cardiol Clin.

[3] Pérez-Molina JA, Molina I (2018). Chagas disease. Lancet.

[4] Barbour K, Miller NH (2008). Adherence to exercise training in heart failure: a
review. Heart Fail Rev.

[5] Lima MM, Rocha MO, Nunes MC, Sousa L, Costa HS, Alencar MC (2010). A randomized trial of the effects of exercise training in Chagas
cardiomyopathy. Eur J Heart Fail.

[6] Nascimento BR, Lima MM, Nunes Mdo C, Alencar MC, Costa HS, Pinto MM (2014). Effects of exercise training on heart rate variability in Chagas
heart disease. Arq Bras Cardiol.

[7] Mediano MF, Mendes FS, Pinto VL, Silva GM, Silva PS, Carneiro FM (2016). Cardiac rehabilitation program in patients with Chagas heart
failure: a single-arm pilot study. Rev Soc Bras Med Trop.

[8] Mendes FSNS, Mediano MFF, Souza FCC, Silva PS, Carneiro FM, Holanda MT (2020). Effect of Physical Exercise Training in Patients With Chagas
Heart Disease (From the PEACH STUDY). Am J Cardiol.

[9] Clark RA, Inglis SC, McAlister FA, Cleland JGF, Stewart S (2007). Telemonitoring or structured telephone support programmes for
patients with chronic heart failure: systematic review and
meta-analysis. BMJ.

[10] Inglis SC, Clark RA, Dierckx R, Prieto-Merino D, Cleland JGF (2015). Structured telephone support or non-invasive telemonitoring for
patients with heart failure (Review). Cochrane Database Syst Rev.

[11] Shaw DK, Heggestad-Hereford JR, Southard DR, Sparks KE (2001). American Association of Cardiovascular and Pulmonary
Rehabilitation Telemedicine Position Statement. J Cardiopulm Rehabil.

[12] Hwang R, Bruning J, Morris NR, Mandrusiakb A, Russell T (2017). Home-based telerehabilitation is not inferior to a centre-based
program in patients with chronic heart failure: a randomised
trial. J Physiother.

[13] Greenhalgh T, Koh GCH, Car J (2020). Covid-19: a remote assessment in primary care. BMJ.

[14] Chambela MDC, Mediano MFF, Carneiro FM, Ferreira RR, Waghabi MC, Mendes VG (2020). Impact of pharmaceutical care on the quality of life of patients
with heart failure due to chronic Chagas disease: Randomized clinical
trial. Br J Clin Pharmacol.

